# Advancing PROTAC Discovery Through Artificial Intelligence: Opportunities, Challenges, and Future Directions

**DOI:** 10.3390/ph18121793

**Published:** 2025-11-25

**Authors:** Kwang-Su Park, Minji Jeon

**Affiliations:** 1 College of Pharmacy, Keimyung University, Daegu 42601, Republic of Korea; parkks@kmu.ac.kr; 2 Department of Biomedical Informatics, Korea University College of Medicine, Seoul 02708, Republic of Korea

**Keywords:** PROTAC, AI, ternary complex prediction, PROTAC molecule design, degradability, ADME prediction

## Abstract

Proteolysis Targeting Chimeras (PROTACs) represent a transformative modality in drug discovery, enabling the selective degradation of disease-relevant proteins through the ubiquitin proteasome system. Despite their therapeutic promise, the rational design of PROTACs remains a complex and resource-intensive process, involving multiple parameters such as target and ligase compatibility, ternary complex formation, linker optimization, and degradation efficiency. Recent advances in artificial intelligence (AI) have provided new strategies to address these obstacles, ranging from structure-based modeling of ternary complexes to degradability prediction, generative linker design, and pharmacokinetic property estimation. This review aims to explore how AI can be leveraged directly or indirectly in the PROTAC development pipeline. First, we analyze existing applications of AI, such as ternary complex structure prediction, degradability prediction, linker design, and ADME prediction. We further discuss how other approaches from the related fields may be adapted to address the challenges of PROTAC discovery. Lastly, we discuss challenges that current AI models face, such as limited data, poor interpretability, and low generalizability. Taken together, overcoming these barriers will enable AI-driven strategies to accelerate PROTAC discovery and provide a more rational framework for targeted protein degrader development.

## 1. Introduction

Many disease-associated proteins, such as scaffolding proteins, transcription factors, or regulatory molecules, lack deep, well-defined binding pockets or catalytic active sites, rendering them inaccessible to traditional occupancy-based pharmacology. These proteins often feature broad, shallow surfaces or intrinsically disordered regions that are poorly suited for high-affinity binding by small molecules [1]. Despite their structural intractability, such proteins frequently play essential roles in cancer, neurodegeneration, and immune disorders, and therefore remain high-value therapeutic targets [2]. To overcome these challenges, targeted protein degradation (TPD) has emerged as a transformative therapeutic strategy that expands the druggable proteome by enabling the modulation of proteins previously considered intractable by conventional small-molecule inhibitors [3,4,5].

**Figure 1:**
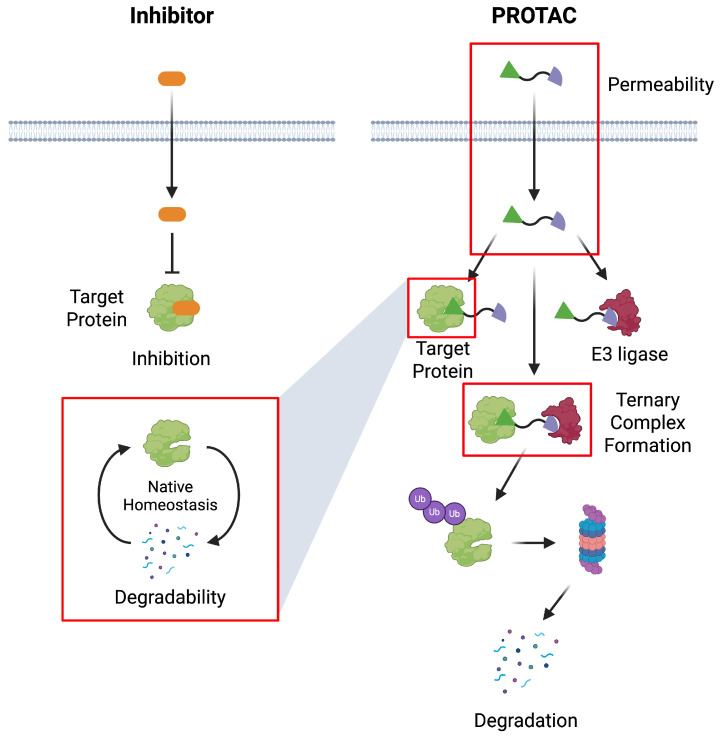
Schematic comparison between conventional inhibition and PROTAC-mediated targeted protein degradation. Unlike small-molecule inhibitors that transiently block target protein activity, PROTACs induce catalytic and event-driven degradation by recruiting an E3 ubiquitin ligase to the target protein, promoting ternary complex formation, ubiquitination, and subsequent proteasomal degradation. Red boxes highlight key considerations in PROTAC discovery, including permeability, target selection informed by native homeostasis–dependent degradability, and ternary complex formation.

Among the most prominent TPD modalities, proteolysis targeting chimeras (PROTACs) represent a class of heterobifunctional small molecules that exploit the cell’s endogenous ubiquitin proteasome system (UPS) to selectively degrade intracellular proteins. PROTACs consist of three key components: a ligand for the protein of interest (POI), a ligand for an E3 ubiquitin ligase, and a linker that connects them. Upon simultaneous engagement of the POI and the E3 ligase, the PROTAC facilitates the formation of a ternary complex that brings the two proteins into close proximity, enabling the ligase to ubiquitinate the POI. This triggers proteasomal recognition and degradation of the target protein. Importantly, the PROTAC molecule is not consumed in this process and can engage in multiple rounds of catalysis, a hallmark of event-driven pharmacology that distinguishes PROTACs from classical inhibitors, which require sustained occupancy of the target at stoichiometric levels [6,7] (Figure 1).

Recent clinical advances have demonstrated the therapeutic potential of this modality. As of 2025, at least 30 PROTACs have entered human clinical trials, with most candidates in phase I or II. Notably, ARV-471 (vepdegestrant), targeting the estrogen receptor (ER), has advanced to phase III clinical trials and is currently under New Drug Application (NDA) labeling, representing the most clinically advanced example of PROTACs in development. These trials not only validate the pharmacological feasibility of targeted protein degradation in humans but also pave the way for the first potential regulatory approvals in this emerging drug class [6,8,9]. Despite their conceptual promise, the rational design of PROTACs remains an inherently complex and resource-intensive endeavor. Achieving efficient and selective protein degradation requires the careful optimization of multiple interdependent parameters. These include the selection of a degradable target protein, the choice of a compatible E3 ligase, the ability to form a structurally stable ternary complex, the positioning and flexibility of the linker, and favorable biophysical properties such as cell permeability, metabolic stability, and intracellular exposure [10]. Notably, many PROTAC candidates fail to induce degradation not due to insufficient binding affinity, but rather because of suboptimal ternary complex geometries that lack cooperativity, or inadequate cellular pharmacokinetics [11,12]. These design bottlenecks make traditional trial-and-error approaches time-consuming and costly, ultimately hindering the pace of PROTAC discovery and development.

In recent years, artificial intelligence (AI) has revolutionized various stages of drug discovery, from de novo molecule generation and target prediction to ADME profiling and structure-based design [13]. However, its application to PROTAC discovery remains nascent. Early studies have demonstrated the potential of machine learning and deep learning in addressing specific subproblems for PROTAC discovery: predicting the likelihood of ternary complex formation, estimating degradability, optimizing linker properties, and modeling compound permeability [14]. As a result, a growing body of research is now exploring how AI-based tools can complement and accelerate PROTAC discovery.

Several excellent reviews have already provided comprehensive overviews of AI applications in general drug discovery and development [15,16,17,18]. Therefore, we do not reiterate those detailed methodologies here. Instead, this review is written from the perspective of how AI specialists can contribute their expertise to PROTAC discovery, which may make certain computational terms less explicitly explained and somewhat challenging for readers from other disciplines. The central aim of this review is to give an overview of the landscape of AI applications in PROTAC discovery and highlight opportunities for future innovation. We begin by reviewing AI models that have already been applied to PROTACs, including ternary complex modeling, degradation prediction, generative models for linker design, and ADME prediction. We also examine other AI models, such as sequence-to-expression models and flow-based generative models, and identify transferable methodologies that could be adapted for PROTAC discovery pipelines. We also discuss major challenges in PROTAC discovery, such as the scarcity of high-quality data, especially experimentally validated ternary complex structures and degradation profiles. In addition, model interpretability and generalizability remain significant concerns, particularly when applying AI tools across diverse target classes and E3 ligase systems. To overcome these barriers, integrated strategies combining physics-informed neural networks, biological validation, and agentic AI frameworks are likely to play a central role.

## 2. PROTAC Discovery Pipeline: Opportunities for AI Integration

**Figure 2:**
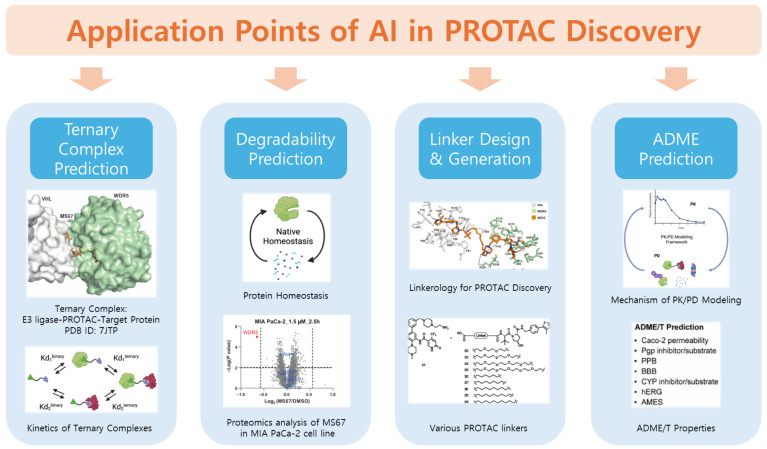
Application points of artificial intelligence (AI) in PROTAC discovery and optimization. AI-based approaches have been applied across multiple stages of PROTAC discovery, including (1) ternary complex prediction, which models the interactions among the target protein, PROTAC, and E3 ligase; (2) degradability prediction, which estimates the degradation potential of target proteins; (3) linker design and generation, enabling de novo or optimized linker construction for improved ternary complex stability and degradation efficiency; and (4) ADME prediction, which supports pharmacokinetic and drug-likeness evaluation through AI–based models of permeability, metabolism, and toxicity. Adapted from Yu et al., Sci. Transl. Med. (2021) [19], licensed under a Creative Commons Attribution 4.0 International License (CC BY 4.0). Modified and combined under the terms of the respective Creative Commons licenses.

The discovery of PROTACs follows a multi-step pipeline that includes target identification, PROTAC design, synthesis, and biological evaluation (Figure 2). This complex workflow presents numerous technical challenges, from selecting appropriate protein targets and E3 ligases to optimizing the ternary complex formation and pharmacokinetic properties of PROTACs. Recent advances in AI offer promising solutions to several bottlenecks throughout this pipeline, enabling more efficient and rational PROTAC discovery.

### 2.1. Overview of the PROTAC Discovery Workflow

The PROTAC discovery process begins with target selection, identifying disease-relevant proteins that are suitable for ubiquitin–proteasome system (UPS)–mediated degradation, as first demonstrated by Sakamoto et al. in their seminal work on PROTAC technology [20]. Following this, E3 ligase selection is a critical step, as the ligase must form a productive ternary complex with both the target protein and the PROTAC molecule. Burslem and Crews provide a comprehensive overview of PROTAC design principles, including iterative linker optimization to maximize ternary complex stability and degradation efficiency [21]. Despite advances in our mechanistic understanding, PROTAC discovery has traditionally relied heavily on a trial-and-error approach. The iterative cycle of synthesis, biological evaluation, and optimization remains resource- and time-intensive, reflecting the complexities inherent in modulating ternary complex formation, target engagement, and cellular permeability. As such, initial PROTAC development is challenged by limited predictive tools and incomplete structural or biophysical data, which often necessitate broad chemical exploration to identify active PROTACs. After synthesis, PROTAC candidates undergo rigorous in vitro and in vivo validation, assessing critical parameters including target degradation potency, selectivity, cellular permeability, and metabolic stability [22]. These sequential steps constitute a feedback-driven workflow, where experimental results inform subsequent design iterations in an ongoing optimization loop.

### 2.2. Critical Bottlenecks: From Target Selection to PROTAC Optimization

Several bottlenecks continue to slow down the PROTAC pipeline:

Target and E3 Ligase Pairing: Although more than 600 E3 ligases are encoded in the human genome, only a small number, most notably VHL and CRBN, are commonly used in PROTAC development. This is largely due to limited structural information, lack of biochemical characterization, and the absence of well-validated small molecule binders [23]. Identifying the most suitable combinations of target proteins and E3 ligases remains a major challenge. There have been efforts to explore alternative E3 ligases beyond VHL and CRBN, such as KEAP1, DCAF16, MDM2, and RNF114. However, these cases are still relatively rare and highly context-dependent. One of the main obstacles is the difficulty in finding small molecules that can bind to these ligases with sufficient potency and selectivity. In addition, E3 ligases show distinct expression patterns depending on the tissues and cell types, which could enable selective degradation in specific biological contexts but also increase the complexity of rational design. Therefore, expanding the usable set of E3 ligases will require continued efforts in ligand discovery, structural analysis, and data-driven profiling of ligase expression and function.

Ternary Complex Formation: The cooperativity and stability of the ternary complex critically influence degradation efficacy. Roy et al. demonstrated the importance of ternary complex structural characterization for rational PROTAC design [24]. Moreover, numerous studies have confirmed that stable ternary complexes substantially contribute to degradation efficiency [19]. Consequently, accurate prediction of ternary complex formation is becoming increasingly important for effective PROTAC development.

Linker and ADMET Optimization: The linker in a PROTAC molecule plays a central role not only in facilitating ternary complex formation between the target protein and E3 ligase but also in modulating critical pharmacokinetic and physicochemical properties. While early-stage PROTAC discovery often employs simple PEG (polyethylene glycol) or alkyl linkers to explore structure–activity relationships [25], it has been consistently shown that even subtle changes in linker length, composition, rigidity, or polarity can dramatically affect both degradation efficacy and drug-like behavior. Importantly, the linker is a major determinant of a PROTAC’s absorption, distribution, metabolism, and excretion (ADME) characteristics. PROTAC molecules typically violate multiple Lipinski’s rules due to their large molecular weight and high flexibility, which frequently result in poor solubility and membrane permeability [4]. Recent work has highlighted that modifications to linker properties, such as reducing hydrogen bond donors, introducing conformational constraints, or minimizing polar surface area, can lead to significant improvements in passive permeability and oral bioavailability. Indeed, most PROTACs that have progressed to clinical trials, including ARV-110 and ARV-471, do not retain their initial screening linkers but instead utilize linkers that have been extensively optimized through medicinal chemistry to strike a balance between degradation potency and favorable pharmacokinetic profiles. This highlights the notion that linker design is not a secondary consideration but a core aspect of PROTAC development strategy. Effective PROTAC optimization, therefore, requires a comprehensive approach that simultaneously addresses ternary complex geometry and ADME properties through rational linker engineering.

Collectively, these challenges highlight that the PROTAC discovery process remains constrained by empirical trial-and-error and incomplete predictive frameworks. Each stage, including target–ligase pairing, ternary complex formation, linker design, and ADME optimization, presents unique computational bottlenecks that limit rational design. These limitations naturally align with areas where artificial intelligence can provide tangible value. In the following section, we therefore examine how emerging AI models directly intervene at each step of this workflow, transforming empirical screening into data-driven, predictive, and generative discovery cycles.

## 3. Current Applications of AI in PROTAC Discovery

Building upon the workflow and bottlenecks outlined in Section 2, AI has begun to play a transformative role in addressing the critical pain points of PROTAC discovery. Specifically, deep learning and generative models have been developed to (i) predict ternary complex formation with atomic precision, (ii) infer degradability from protein–ligase–compound context, (iii) rationally generate and optimize linkers, and (iv) model pharmacokinetic behavior of large heterobifunctional molecules. These tools collectively mark the transition from descriptive to predictive modeling of degrader design. In this section, we summarize the representative AI approaches specifically developed for PROTACs and highlight their distinctive contributions to the field (Table 1).

### 3.1. Ternary Complex Prediction

Accurate prediction of ternary complexes is a fundamental requirement in PROTAC research, as productive degradation depends on the formation of stable and cooperative assemblies between the target protein, the E3 ligase, and the PROTAC. However, it remains challenging due to the conformational flexibility of linkers and the cooperative nature of E3–target interactions. The earliest systematic efforts to model PROTAC-induced ternary complexes were demonstrated by Drummond and Williams [42]. Using Monte Carlo conformational sampling combined with docking, they showed that it was possible to recover a small number of near-native poses. However, their framework showed limited accuracy in ranking, highlighting the need for improved sampling and scoring strategies. PRosettaC [43] integrates global docking and Rosetta-based refinement under PROTAC-derived distance constraints, recovering near-native ternary structures for BRD4 and BTK and enabling rationalization of experimental structure–activity relationships. While effective for well-studied systems, its reliance on classical physics-based docking limits accuracy and generalizability across diverse targets.

**Figure 3:**
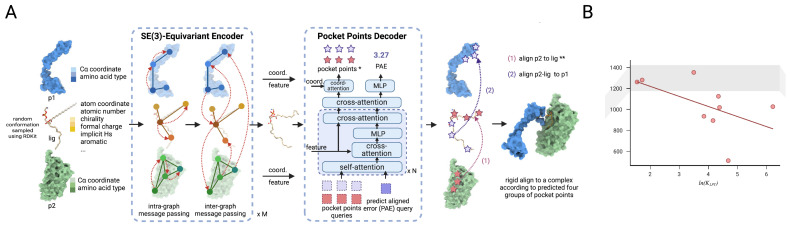
Overview of the DeepTernary model and its structure–property correlation analysis for BRD4–VHL ternary complexes. (**A**) Schematic representation of DeepTernary, an SE(3)-equivariant graph neural network architecture with attention blocks. * For PROTAC, the pocket points are derived from unbound structures, don’t need to be predicted. ** For MG(D), the ligand (lig) and POI (p2) are simultaneously aligned to E3 ligase (p1). (**B**) Correlation analysis between the predicted buried surface area (BSA) and degradation potency scores ($ln(K_{LPT})$) for the modeled BRD4–VHL ternary complexes assembled with various PROTACs. Adapted from Xue et al., Nature Communications (2025) [26], licensed under a Creative Commons Attribution 4.0 International License (CC BY 4.0). Modified and combined under the terms of the respective Creative Commons licenses.

Recently, deep learning has been leveraged to overcome the limitations of physics-based docking. For example, AlphaFold3 [27] has been applied to ligand-mediated ternary complex prediction, showing that it improves interface prediction accuracy relative to purely docking-based methods. However, AlphaFold3 is primarily a general-purpose protein–ligand predictor, and while it provides structural hypotheses, it was not specifically optimized to capture the cooperativity or degradation potency unique to PROTAC-mediated ternary complexes. By contrast, DeepTernary [26], an SE(3)-equivariant graph neural network developed for end-to-end prediction of ternary complex structures, was trained on over 20,000 experimentally resolved ternary complexes collected from the PDB [44]. Although these ternary complexes are not specific to PROTAC systems, they capture general principles of protein-to-protein association that are relevant to degrader design, because PROTACs act by inducing new and productive protein-to-protein interactions between the target and the E3 ligase rather than merely bringing them into spatial proximity. The model takes as input the unbound structures of the target protein and E3 ligase, along with the molecular graph of the degrader, and predicts the 3D ternary configuration through inter- and intra-graph attention–based message passing and an attention-based decoder (Figure 3A). With the SE(3)-equivariant design, it ensures that geometric relationships are preserved under rotation and translation, enabling physically consistent learning of spatial interactions. When benchmarked on curated ternary datasets, DeepTernary achieved substantially lower interface RMSD and higher DockQ scores than AlphaFold3, indicating superior recovery of native ternary geometries. More importantly, the model reveals a quantitative correlation between predicted buried surface area (BSA) and degradation potencies estimated from $K_{LPT}$ constants (Figure 3B). This indicates that the geometric complementarity learned by the model reflects real degradability trends, supporting its potential use in high-throughput in silico screening and prioritization of degrader candidates. However, the generalizability of these predictions across diverse ligase–target pairs remains unproven, as most datasets are skewed toward well-studied systems like BRD4–VHL. Overfitting to structurally similar training complexes is a small potential risk, given the small number of high-resolution ternary complex structures available.

### 3.2. Degradability Prediction

**Figure 4:**
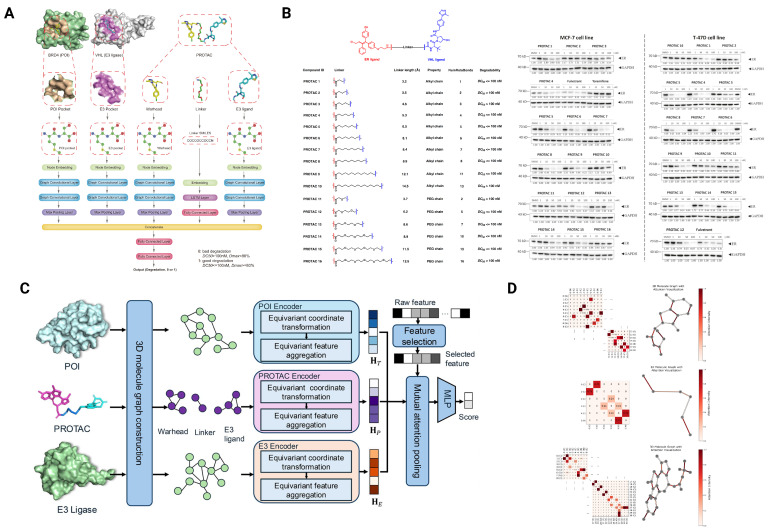
Overview of the DeepPROTACs and DegradeMaster frameworks and their results for PROTAC degradability prediction. (**A**) The network architecture of DeepPROTACs, a neural network model integrating graph- and sequence-based representations of targets, E3 ligases, ligands, and linkers to predict PROTAC degradation activity. (**B**) Chemical structures and physicochemical properties of 16 PROTACs in the experimental dataset, along with Western blotting analyses and densitometry quantifications of ER protein levels following treatment with the corresponding PROTACs. (**C**) The overall framework of DegradeMaster, a semi-supervised, E(3)-equivariant graph neural network that integrates 3D structural information and pseudo-labeling to predict PROTAC degradability. (**D**) Visualization of attention weights for the PROTAC molecule (PROTAC-DB ID: 194), illustrating the key molecular features contributing to BRD2 degradation via CRBN E3 ligase recruitment. Adapted from Li et al., Nature Communications (2022) [28], and Liu et al., Bioinformatics (2025) [31], licensed under the Creative Commons Attribution 4.0 International License (CC BY 4.0). Modified and combined under the terms of the respective Creative Commons licenses.

While ternary modeling captures structural feasibility, degradation prediction addresses a distinct question: whether a designed PROTAC will actually trigger efficient target ubiquitination and proteasomal clearance inside cells. This task goes beyond structural compatibility, requiring integration of molecular features, binding context, and cellular factors. It is worth noting that a target protein’s native homeostasis, including its intrinsic turnover rate, stability mechanisms, and proteostatic regulation, plays a decisive role in degradability. Proteins that are strongly buffered by cellular quality control systems or maintained through tightly regulated stability networks often resist PROTAC-induced degradation even when ternary complex formation is structurally favorable. To facilitate such studies, PROTAC-DB [45] provides a comprehensive resource of over 6000 PROTACs with some annotated $DC_{50}$ and $D_{max}$ values, enabling quantitative modeling of degradation outcomes. Using this resource, DeepPROTACs [28] introduced one of the first deep learning frameworks for degradation prediction. The model combines molecular graph embeddings of PROTACs with protein- and E3-specific sequence features to predict whether a compound induces high or low degradation (Figure 4A). When benchmarked on multiple targets, DeepPROTACs achieved a classification accuracy of 78%, and importantly, the authors validated predictions experimentally, correctly identifying 11 out of 16 PROTACs as active degraders(Figure 4B), confirming the model’s ability to prioritize active degraders. Subsequent studies by Ribes et al. extended this approach by incorporating pre-trained embeddings with semi-supervised learning to improve degradation activity prediction under limited labeled data, while also evaluating model generalization to unseen compounds and protein targets [29]. Complementing these molecule-centric models, the MAPD framework [30] predicts the intrinsic degradability of proteins independent of specific PROTACs. It models protein-intrinsic determinants such as ubiquitination motifs and solvent-exposed lysines, achieving an AUROC of around 0.78 for kinase degradability and showing potential applicability to non-kinase targets.

More recent methods focus on incorporating 3D geometry and interpretability. DegradeMaster [31] employs an E(3)-equivariant graph neural network that integrates spatial molecular information with a semi-supervised learning scheme, allowing it to leverage unlabeled PROTAC data from PROTAC-DB (Figure 4C). The model not only reaches high predictive accuracy, with an AUROC of up to 0.88, but also provides attention-based explanations that highlight key atoms most strongly influencing degradation probability. Visualization of these attention maps reveals that atoms at the linking area connecting the warhead- and E3- binding ligand and binding regions of the POI and E3 ligase show higher attention scores, which is consistent with experimentally observed structure–activity trends (Figure 4D). Separately, PrePROTAC [32] applied interpretable machine learning at a genome-wide scale, highlighting previously understudied proteins as potential PROTAC targets, thereby expanding the druggable space. Together, these efforts illustrate how AI can move beyond classification toward biologically meaningful prioritization of substrates. Despite promising results, most models suffer from limited generalizability to unseen targets, particularly non-kinase or non-CRBN substrates. Cell-line specificity and expression context are often underrepresented in training, leading to false positives or negatives in diverse biological settings. In addition to biological diversity, inconsistencies in experimental quantification further affect model reliability. Although standardized metrics such as $DC_{50}$ and $D_{max}$ are conceptually defined to measure degradation potency, in practice their determination varies widely across studies in terms of assay format, time point, and cell type. This lack of harmonization makes it difficult to establish uniform ground-truth labels and can inflate apparent model performance. As a result, even well-performing models should be interpreted within the context of underlying data uncertainty, emphasizing the need for standardized quantitative benchmarks for PROTAC degradation.

### 3.3. Linker Design and Optimization

The linker is the chemical bridge that connects the warhead and the E3 ligase ligand in a PROTAC molecule. Its length, flexibility, and geometry critically determine whether the two proteins can form a productive ternary complex. Because experimental linker optimization often requires numerous synthetic iterations, AI–based generative models have become powerful alternatives for rational design.

**Figure 5:**
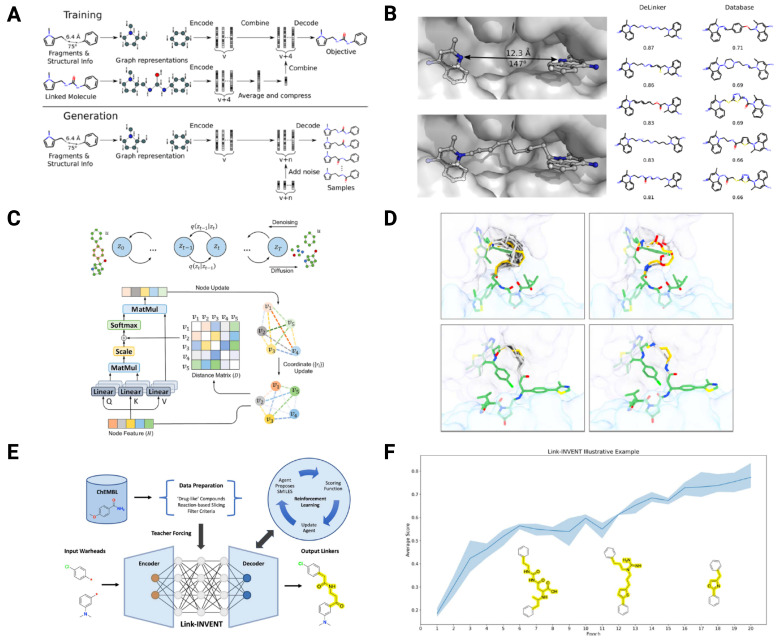
Comprehensive overview of deep generative frameworks for linker/PROTAC generation in DeLinker, DiffPROTACs, and Link-INVENT models. (**A**) Illustration of training and generation procedures demonstrating the overall workflow of model optimization and molecular sampling of DeLinker. (**B**) Comparison of DeLinker with an exhaustive database search, highlighting improvements in linker similarity and diversity. (**C**) Overview of the DiffPROTACs diffusion-based architecture for generative modeling of PROTAC linkers and optimization-guided generation. (**D**) Case studies showing representative BRD4–VHL PROTACs and the corresponding linkers generated by DiffPROTACs. (**E**) Training and inference overview of Link-INVENT, describing the reinforcement learning–based fine-tuning strategy for linker generation. (**F**) Illustrative example of Link-INVENT demonstrating its improvement over epochs to design linkers for given objectives. Adapted from Guo et al., Digital Discovery (2023, CC BY 3.0) [35], Imrie et al., J. Chem. Inf. Model. (2020, CC BY) [33] and Li et al., Briefings in Bioinformatics (2024, CC BY-NC 4.0) [38]. Modified and combined under the terms of the respective Creative Commons licenses.

Graph-based generative models such as DeLinker [33] build linkers atom-by-atom in three-dimensional space. DeLinker adopts a conditional graph variational autoencoder (VAE) architecture that simultaneously models both the chemical topology and the 3D spatial coordinates of atoms. In this framework, each molecular fragment is encoded into a latent vector that captures local chemical environments and geometric constraints, including the distance and angle between exit vectors. During decoding, the model sequentially generates new atoms and bonds conditioned on these geometric features, ensuring that the synthesized linker maintains the correct orientation and avoids steric clashes. This allows DeLinker to grow linkers directly in 3D space while preserving realistic bond lengths and angles (Figure 5A). As shown in the comparison with exhaustive database searches, DeLinker successfully recovered highly similar linkers with a 3D similarity greater than 0.85 to the reference linker, while the database baseline, which randomly sampled linkers from existing molecules, failed to generate comparable results (Figure 5B). This demonstrates the model’s ability to infer plausible 3D connections between fragments. In addition, 3DLinker [34] incorporates E(3)-equivariant latent spaces to have a precise atom-level description of molecule geometry and to ensure generated conformers remain consistent with structural constraints.

Moreover, diffusion-based approaches have also emerged, including DiffPROTACs [38], which adapt denoising diffusion to PROTAC generation, learning to generate entire degrader molecules from noise while maintaining structural coherence between the warhead and E3 ligand (Figure 5C). Its O(3)-equivariant graph transformer ensures rotational invariance, and fine-tuning on a curated PROTAC dataset achieved a 93.9% chemical validity rate. The spatial structure of the generated linkers closely resembles the crystal structures (Figure 5D). Moreover, DAD-PROTAC [39] is a domain-adapted diffusion model that corrects for chemical space mismatch between small molecules and PROTACs via density ratio estimation, enabling efficient fine-tuning and high-quality linker generation under limited PROTAC data.

Reinforcement learning (RL) has also been widely applied to linker optimization. Link-INVENT [35] combines SMILES-based generative modeling with RL to optimize multiple molecular properties simultaneously, such as solubility, flexibility, and hydrogen-bonding capacity. As training progresses, the agent learns to generate linkers that maximize a user-defined multi-objectives (Figure 5E). The result shows a steady improvement in the average objective score across epochs as the model learns to connect two benzene rings with two objectives of minimizing hydrogen bond donors and maintaining a single-ring linker structure (Figure 5F). PROTAC-RL [37] integrates transformer-based linker generation with memory-assisted RL to design viable linkers while optimizing pharmacokinetic profiles even under data-scarce conditions by pre-training on a quasi-dataset followed by fine-tuning on real PROTAC data. Moreover, ShapeLinker [36], an RL method coupled with fast attention-based point cloud alignment, has been introduced to directly optimize linker geometry in three-dimensional space. These innovations collectively highlight a shift from empirical trial-and-error to rational, property-aware linker engineering that can simultaneously account for structural fit and drug-like properties.

### 3.4. ADME Properties Prediction

PROTACs frequently fall outside conventional drug-likeness rules, creating persistent challenges for ADME properties. Early modeling efforts relied on molecular descriptors and classical classifiers such as decision trees, random forests, and support vector machines [41]. Applied to CRBN- and VHL-based PROTACs, these approaches achieved predictive accuracies above 80% for cell permeability, identifying lipophilicity and molecular size as dominant determinants. Although these studies demonstrated promising accuracy, their scope was limited to relatively small and homogeneous datasets focused on permeability. In practice, comprehensive ADME modeling must extend beyond permeability to include solubility, metabolic stability, and enzyme interactions, and this is particularly challenging because PROTAC-specific datasets remain scarce. Addressing this data limitation, Peteani et al. systematically evaluated quantitative structure–property relationship (QSPR) models for predicting ADME-related properties of PROTACs and molecular glues, including permeability, metabolic clearance, cytochrome P450 inhibition, plasma protein binding, and lipophilicity [40]. Their key contribution was to pretrain multi-task models on large-scale small molecule datasets and then fine-tune them on degrader data, effectively leveraging transfer learning to overcome data scarcity. This approach markedly improved predictive performance, achieving misclassification errors below 4% for molecular glues and around 15% for PROTACs. These results indicate that while classical classifiers can achieve strong performance in narrow tasks, transfer learning is critical to scale predictive modeling across the broader and more data-sparse chemical space of PROTACs. Nevertheless, ADME and permeability data for degraders remain highly heterogeneous, as assay conditions, platforms, and cell lines differ substantially across studies. As a result, even models showing high predictive accuracy may be constrained by experimental variability, underscoring the need for community-wide efforts to unify and standardize such datasets. Also, it should be noted that the model reported in [40] was trained on proprietary, non-public datasets, which limits the possibility for external validation and comparison. Nevertheless, such industrial datasets have provided valuable internal insights, and continued efforts toward transparent and shareable degrader data will be crucial to ensure reproducibility and wider applicability.

While current AI applications have demonstrated clear value across specific stages of PROTAC design, most remain specialized and operate in isolation. The next frontier lies in integrating these task-specific models into unified, multi-modal frameworks capable of reasoning across sequence, structure, and cellular response. Section 4, therefore, explores emerging and transferable AI paradigms—from protein language models to diffusion and flow-based generators—that could bridge these silos and establish a cohesive foundation for end-to-end, AI-enabled PROTAC discovery.

## 4. Emerging and Transferable AI Models for PROTAC Discovery

**Figure 6:**
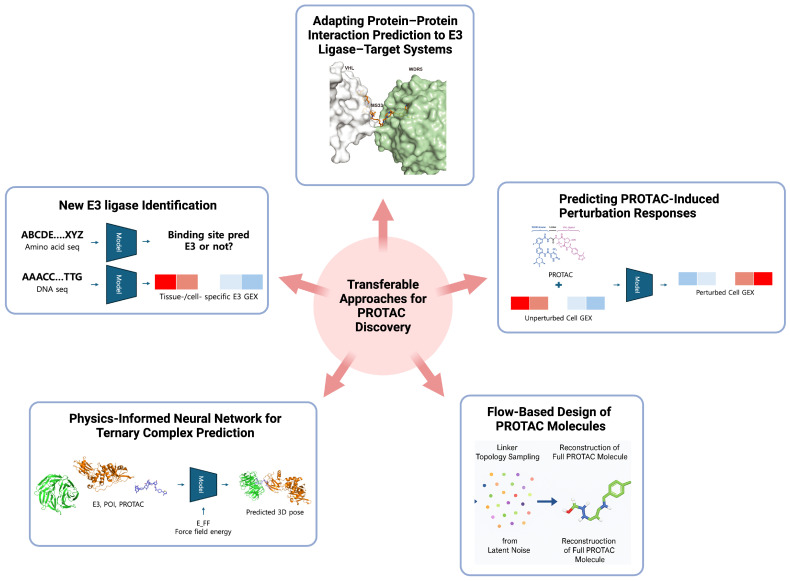
Next-generation AI frameworks for PROTAC discovery, including (1) adaptation of protein–protein interaction prediction models to E3 ligase–target systems for accurate estimation of interfacial residues and binding cooperativity, (2) prediction of PROTAC-induced transcriptomic perturbations by linking chemical structure with gene expression response profiles, (3) flow-based generative models for de novo PROTAC molecule design, (4) physics-informed neural networks for ternary complex prediction incorporating energy-based constraints or 3D equivariant learning for pose generation, and (5) amino acid or genomic information-based identification of tissue- or cell-specific E3 ligases. Adapted from Yu et al., Sci. Transl. Med. (2021) [19], licensed under a Creative Commons Attribution 4.0 International License (CC BY 4.0). Modified and combined under the terms of the respective Creative Commons licenses.

Alongside the rapid progress of PROTAC-specific AI models, recent advances in AI models from related areas of biology and chemistry offer valuable opportunities for PROTAC design. These transferable technologies, ranging from sequence-based foundation models to chemical perturbation response prediction frameworks, could significantly accelerate PROTAC research if adapted appropriately. This section highlights promising methods from related fields and identifies areas where dedicated PROTAC-specific model development is still required (Figure 6).

### 4.1. Sequence- and Transcriptome-Based E3 Ligase Identification

One of the major bottlenecks in PROTAC discovery is the narrow reliance on a handful of E3 ligases such as CRBN and VHL, despite the human genome encoding more than 600 ligases with potential therapeutic utility. This bias largely reflects the scarcity of well-characterized ligands and the lack of structural and biochemical data for most ligases. Recent advances in AI now offer a means to systematically explore and prioritize E3 ligases by integrating diverse sequence- and transcriptome-based biological signals. At the protein level, protein language models (PLMs) such as ESM-2 [46] and ProtTrans [47], trained on massive sequence corpora, provide rich embeddings that capture structural and functional properties of proteins. When applied to the full human genome, these embeddings enable unsupervised clustering of E3 ligases with shared ubiquitination domains and prediction of substrate-recognition motifs or binding pockets that have not been experimentally annotated. Such representations can also guide the de novo design of ligase-binding motifs or even entirely new E3 ligases with desired substrate specificity, by incorporating them into generative protein design frameworks. At the genomic level, sequence-to-expression models including ExPecto [48], Enformer [49], Borzoi [50] and AlphaGenome [51] can complement PLM-based predictions by estimating tissue- and cell-type–specific E3 expression directly from genomic DNA. These models learn regulatory sequence grammars that map promoter and enhancer sequences to quantitative expression patterns across thousands of tissues. Incorporating their predictions allows ranking of E3 ligases not only by biochemical plausibility but also by their predicted availability in disease-relevant cellular contexts. Integrating these approaches with ligandability resources such as ELIOT [52], UbiHub [53], and E3Atlas [54] provides a rational pipeline to expand the E3 ligase repertoire, moving beyond empirical selection toward context-aware, sequence-driven discovery of novel ligases suitable for PROTAC design.

### 4.2. Predicting Ligase–Substrate Specificity

In PROTAC design, it is important to predict which E3 ligases can productively engage specific substrates within a given cellular environment [55]. Recent advances in protein–protein interaction (PPI) prediction have provided transferable strategies to model E3–substrate recognition in a more quantitative and spatially resolved manner. For instance, geometric deep learning frameworks such as MaSIF [56] learn residue-level surface representations, providing structural determinants that guide ligase selection. Another model named MAPE-PPI [57] introduces a microenvironment-aware protein embedding framework that represents each residue together with its local three-dimensional and sequential neighborhood, enabling accurate and scalable PPI prediction with an effective balance between efficiency and accuracy. Adapting these architectures to E3 ligase systems will require incorporating additional biological context, such as degron accessibility, ubiquitin-transfer geometry, and lysine spatial distribution to predict productive ubiquitination.

### 4.3. Flow-Based Generative Modeling

Generative modeling frameworks, originally developed for molecular and protein design, can also be applied to PROTAC discovery. Specifically, some diffusion models have demonstrated impressive ability to generate chemically valid and geometrically consistent molecules, including complex scaffolds and linkers [38,39]. Recently, flow matching models [58] have emerged as an efficient alternative to diffusion models, learning continuous transformations from a base distribution, such as Gaussian noise, to the complex data distribution. This approach enables faster convergence during training and produces more stable samples. These methods have already been adapted for molecule and protein design tasks, enabling efficient generation of property-constrained molecules and 3D conformations [59,60]. In the context of PROTACs, flow matching could be leveraged to condition generation explicitly on ternary pocket geometries or linker exit vectors, while simultaneously enforcing physicochemical constraints such as lipophilicity, polar surface area, or permeability. Beyond linker optimization, such frameworks also offer the possibility of designing entirely new warheads for emerging targets or ligands for E3 ligases to expand the PROTAC toolbox, thereby broadening the chemical and biological space accessible for targeted protein degradation. Moreover, flow-matching–based generative surrogates [61,62], initially developed to accelerate molecular dynamics simulations, offer a scalable way to approximate the conformational ensembles underlying these interactions. Together, these approaches could enable large-scale in silico screening of ligase–substrate specificity by combining high-resolution geometric recognition with efficient ensemble-level sampling.

### 4.4. Physics-Informed Neural Networks

Purely data-driven neural networks, especially when trained on limited datasets, struggle to internalize fundamental physical relationships, such as including steric repulsion, bond connectivity, or force-field energetics, whereas fully atomistic simulations are too computationally expensive for large-scale applications. A promising intermediate solution is physics-informed neural networks that embed molecular mechanics directly into their architectures or loss functions [63,64]. By constraining representations with physically meaningful terms, these models can capture the energetic landscape of PROTAC assemblies while retaining scalability. Adapting such approaches to PROTAC systems could yield neural network models that respect fundamental molecular mechanics while remaining data-efficient—providing energy-consistent predictions of ternary structure prediction without the need for exhaustive atomistic simulations.

### 4.5. Transcriptome-Based Modeling of Chemical Perturbation Responses

While ternary complex modeling captures the structural feasibility of PROTAC action, it does not provide insight into the broader cellular consequences of PROTAC treatment. A similar challenge has long existed in traditional inhibitor development, where structural docking or binding assays alone could not explain compound-specific transcriptional outcomes. To address this gap, models trained on large-scale perturbational transcriptome datasets such as ConnectivityMap [65] and Tahoe-100M [66], which are primarily derived from inhibitor-induced perturbations, have been widely used to predict how chemical treatments change gene expression levels. These resources, which couple millions of chemical perturbations to matched transcriptional readouts, enable the in silico prediction of chemical perturbation response [67,68]. For PROTACs, a similar modeling paradigm could be adopted once PROTAC-specific perturbational datasets become available, allowing prediction of how targeted protein degradation reshapes transcriptomic states. Such models could be used to (i) prioritize PROTAC molecules that yield the desired transcriptomic fingerprint, (ii) filter out designs predicted to trigger toxicity-associated signatures. In this way, perturbation-response modeling complements structural and ADME predictions by providing a system-level view of PROTAC efficacy and safety, accelerating the triage of PROTAC candidates before entering costly experimental assays.

Taken together, these diverse AI paradigms, ranging from sequence-based protein language models and transcriptome predictors to diffusion and flow-based molecular generators, represent complementary layers of an emerging ecosystem for degrader design. Sequence and transcriptome models expand the accessible ligase and substrate space, while geometric and physics-informed networks enhance ternary modeling fidelity, and generative models accelerate the creation of linker and ligand candidates with desired pharmacological traits. Their convergence defines a unified vision in which multi-modal AI frameworks collectively address the fundamental bottlenecks of PROTAC discovery, paving the way toward integrated, adaptive, and experimentally grounded AI-driven PROTAC design.

## 5. Challenges and Limitations of Current AI Approaches

Despite rapid advances in AI-driven PROTAC discovery, several fundamental challenges limit the robustness, interpretability, and translational impact of current models. These limitations are not unique to PROTACs but are amplified by the unique structural complexity, pharmacological liabilities, and data sparsity of the PROTAC chemical space. Below, we discuss the main challenges that remain to be overcome for the effective integration of AI into PROTAC design.

### 5.1. Data Scarcity and Standardization in PROTAC Datasets

Public PROTAC resources remain limited both in scale and annotation quality. For example, PROTAC-DB [45] and TPDdb [69] contain several thousand to ten thousands entries, yet only a fraction are associated with standardized, quantitative degradation metrics such as $DC_{50}$ or $D_{max}$. However, their assay conditions differ substantially across studies in terms of cell lines, exposure times, ligase context, and detection methods [4,70]. Such heterogeneity reduces data comparability and introduces noise into model training [71]. In addition, experimentally resolved ternary complex structures remain scarce, with fewer than one thousand entries currently available, leaving most compounds without corresponding 3D interaction data. The limited size of curated datasets increases the risk of overfitting and reduces reproducibility [72]. Addressing these issues will require larger, standardized datasets developed through community-wide benchmarking efforts. Such standardization is not only essential for data comparability but also directly determines the reliability of AI models discussed in Section 3. In addition, some recent AI models rely on proprietary datasets that are not publicly available. While such data have accelerated progress within individual organizations, their limited accessibility makes it difficult for the broader community to independently verify or benchmark model performance. Continued collaboration and gradual sharing of standardized degrader datasets will be important for improving reproducibility and community engagement.

### 5.2. Limited Generalization Across Targets and Ligases

Most AI models trained on PROTAC data show strong performance in retrospective benchmarks but show sharp performance declines when applied to new targets or less-studied ligases [71,72]. This reflects the inherent bias in available data, which is heavily skewed toward a few popular ligases, such as CRBN and VHL and a limited number of protein targets [4,70]. Generalization to other E3 ligases, substrates, or therapeutic contexts remains a major unsolved problem [23]. Without addressing this bias, models risk reinforcing the current narrow scope of PROTAC discovery rather than enabling expansion into unexplored ligase–target combinations. Transfer learning, domain adaptation, and multi-task modeling across chemical modalities may provide paths forward, but systematic validation across diverse ligase–substrate combinations is still lacking.

### 5.3. Lack of Interpretability

Many current approaches operate as black-box predictors, achieving high accuracy without offering mechanistic insight. Degradability classifiers rarely reveal which sequence or structural features drive predictions, while ternary complex models often generate plausible poses without clarifying determinants of cooperativity or ubiquitination efficiency. This opacity limits both scientific trust and translational utility. Moreover, relatively few AI-designed PROTACs have progressed to prospective wet-lab validation, meaning that much of the current evidence remains computational. Improving interpretability through attention mechanisms, feature attribution, or physics-informed modeling, combined with systematic biological validation, is necessary to bridge the gap between prediction and mechanism.

### 5.4. The Missing Experimental Feedback Loop

Finally, AI-driven PROTAC design has yet to be fully integrated into iterative experimental pipelines. Unlike traditional drug discovery, where design–synthesis–testing loops are standard, most PROTAC models operate in isolation from real-time experimental feedback. This prevents models from learning from their own failures and refining predictions dynamically. Embedding AI into closed-loop workflows, where predictions guide synthesis and results are immediately fed back into model training, will be critical to overcome data scarcity, validate predictions, and accelerate optimization. Early demonstrations of lab-in-the-loop drug design suggest that such integration could be transformative for PROTAC discovery [73].

## 6. Future Perspectives: Toward AI-Driven PROTAC Design

AI is transforming drug discovery, yet its tangible impact differs greatly across modalities. In the small-molecule domain, AI has already delivered practical success. For example, Insilico Medicine developed the first end-to-end AI-designed small-molecule drug, rentosertib, a TNIK inhibitor for idiopathic pulmonary fibrosis that advanced to phase II clinical trials in 2024 after being identified, optimized, and nominated entirely through AI-driven generative design and preclinical modeling [74]. Comparable AI frameworks are now routinely employed in major pharmaceutical pipelines for target identification, de novo molecule generation, and ADME optimization.

By contrast, the application of AI to targeted protein degradation remains at an earlier stage. To date, no PROTACs in clinical trials have originated directly from AI-based molecular design. Nonetheless, several industrial collaborations indicate that this transition is already underway. VantAI, in partnership with Bristol Myers Squibb, Janssen, and other global pharmaceutical companies, is integrating deep generative and reinforcement-learning models to accelerate the design of degraders and molecular glues. Such initiatives exemplify how AI-assisted PROTAC discovery is evolving from conceptual modeling to early industrial implementation, linking computational design with experimental validation.

For these approaches to mature, data accessibility and feedback integration are essential. Much of the existing degrader data remains fragmented or proprietary, limiting reproducibility and cross-benchmarking. Establishing a pre-competitive data-sharing initiative through expanded resources such as PROTAC-DB and TPDdb would provide the transparency required for rigorous evaluation of AI models and facilitate their translation into clinically actionable degraders. Collaborative data ecosystems between academia, startups, and large pharmaceutical organizations could therefore serve as the foundation for the next generation of AI-driven PROTAC platforms.

For AI to transition from predictive modeling to practical design, it also must be tightly integrated with experimental workflows. Current PROTAC pipelines rarely incorporate continuous feedback, preventing models from learning from both successes and failures. The next step is to embed AI into adaptive closed-loop systems in which predictions guide synthesis and high-throughput assays, and the resulting data are immediately fed back into model refinement. Building on this foundation, AI agents offer a way to coordinate such workflows. Agents are autonomous systems that connect multiple models, tools, and datasets, dynamically deciding which ligase–substrate pairs to explore, which linkers to propose, and which assays to run. By incorporating experimental feedback, they convert otherwise static pipelines into adaptive, self-improving design frameworks. The long-term vision is a fully automated PROTAC design platform where a therapeutic target is specified and an optimized PROTAC emerges as output. Such a system would unify target prioritization, ligase selection, ternary complex modeling, linker generation, and ADME optimization into a single computational workflow [75]. Coupled with iterative retraining and prospective validation, this paradigm would represent a decisive shift from heuristic, trial-and-error discovery to systematic, AI-driven PROTAC design.

## Figures and Tables

**Table 1:** AI models applied in PROTAC discovery. Abbreviations: ADME, absorption, distribution, metabolism, and excretion; BSA, buried surface area; DNN, deep neural network; GNN, graph neural network; kNN, k-nearest neighbors; MLP, multi-layer perceptron; MTL, multi-task learning; PK, pharmacokinetics; PLM, protein language model; POI, protein of interest; PPI, protein–protein interaction; PTM, post-translational modification; QSPR, quantitative structure–property relationship; RF, random forest; RL, reinforcement learning; RNN, recurrent neural network; SMILES, simplified molecular input line entry system; VAE, variational autoencoder.

Application Area	Model Name	Architecture	Input	Output	Key Features	Ref.
Ternary Complex Prediction	DeepTernary	SE(3)-equivariant GNN	PROTAC molecular graph + POI/E3 pocket graph with 3D coordinates	3D ternary complex	Predicted BSA indicates degradability	[26]
	AlphaFold3	Diffusion Transformer	Protein sequence + ligand SMILES	3D ternary complex	Diffusion-based multimodal model predicting 3D complexes of proteins, ligands, and nucleic acids	[27]
Degradability Prediction	DeepPROTACs	GNN + RNN + MLP	PROTAC molecular graph + POI/E3 pocket graph	PROTAC degradability (high/low)	Joint molecule–protein modeling for degradation prediction	[28]
	Ribes et al.	Pretrained embedding models + linear classifier	PROTAC SMILES + POI/E3 sequence + cell-line metadata	PROTAC degradability (high/low)	Incorporated cell line context for degradation prediction	[29]
	MAPD	RF	Protein features (PTMs, PPI, length, etc.)	Protein degradability (tractable/ non-tractable)	Protein-level degradability prediction from intrinsic features	[30]
	DegradeMaster	E(3)-equivariant GNN	3D molecule graphs of PROTAC and POI/E3	PROTAC degradability (high/low)	Mutual-attention pooling and pseudo-labeling	[31]
	PrePROTAC	RF	PLM embedding of protein sequence	CRBN-specific protein degradability	Protein-level degradability prediction and key residues identification by eSHAP	[32]
Linker Design & Generation	DeLinker	GNN	Anchor fragments (warhead + E3 ligand) in graph with 3D structural info	3D linker structures	Distance/angle constrained fragment linking	[33]
	3DLinker	E(3)-equivariant graph VAE	Anchor fragments (warhead + E3 ligand) in graph with 3D coordinates	3D linker structures	generates physically consistent linkers with accurate spatial alignment	[34]
	Link-INVENT	RNN + RL	Anchor fragments (warhead + E3 ligand) as SMILES	Optimized linker SMILES	Multi-parameter optimization	[35]
	ShapeLinker	RNN + RL with 3D point cloud alignment	Anchor fragments (warhead + E3 ligand) as SMILES	Optimized linker SMILES	Geometry-conditioned method based on Link-INVENT	[36]
	PROTAC-RL	Transformer + RL	Anchor fragments (warhead + E3 ligand) as SMILES	Full PROTAC molecules with generated linker	Pretrained on quasi-PROTACs; RL optimizes PK; prospective validation	[37]
	DiffPROTACs	Diffusion + O(3)-equivariant graph Transformer	Anchor fragments (warhead + E3 ligand) as molecular graphs with 3D coordinates	Full PROTAC molecules with generated linker	Diffusion refines noisy linker atoms into valid 3D structures; high validity and structural realism	[38]
	DAD-PROTAC	Domain-adapted diffusion	Anchor fragments (warhead + E3 ligand) as molecular graphs with 3D coordinates	Full PROTAC molecules with generated linker	Corrects distribution gap between small molecules and PROTACs via density-ratio guided score adjustment; efficient fine-tuning and reduced overfitting	[39]
ADME & Permeability	Peteani et al.	MTL GNN + DNN	PROTAC SMILES	ADME-related properties (solubility, permeability, stability, etc.)	Used transfer learning to adapt QSPR models to degraders	[40]
	Poongavanam et al.	kNN + RF	17 physicochemical descriptors	Permeability classes	Size and lipophilicity dominate; strong blind accuracy on VHL set	[41]

## Data Availability

No new data were created or analyzed in this study.
